# Corrigendum to: Exposure‐safety analysis of QTc interval and transaminase levels following bedaquiline administration in patients with drug‐resistant tuberculosis

**DOI:** 10.1002/psp4.13005

**Published:** 2023-06-25

Tanneau, L, Svensson, EM, Rossenu, S, Karlsson, MO. Exposure–safety analysis of QTc interval and transaminase levels following bedaquiline administration in patients with drug‐resistant tuberculosis. *CPT Pharmacometrics Syst Pharmacol*. 2021;10:1538–1549. doi:10.1002/psp4.12722

In the published version of the above article, the authors' noticed an error in Figure 1.

Figure 1 illustrates the different components of the final structural QTcF model. As stated in the legend, the figure is supposed to illustrate the typical profile of QTcF interval over time without comedication (full line) as well as the typical profile of the drug effect (dotted line), the time effect (dot‐dashed line) and the baseline QTcF0 (dashed line). The circadian rhythm is depicted by the shaded area around the typical profile of QTcF interval. However, we noticed that those profiles were mistakenly generated including inter‐individual variability, thus the original figure does not show the typical profiles.

The corrected figure is shown below:

**FIGURE 1:**
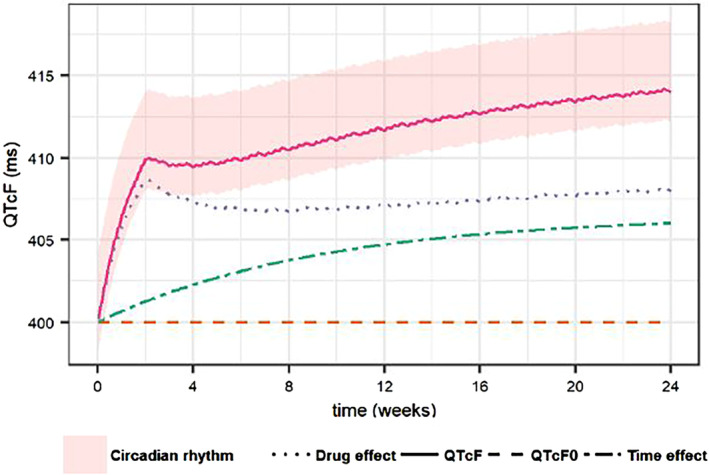
Illustration of the typical profile of QTcF interval over time without co‐medication (full line) as well as the typical profile of the drug effect (dotted line), the time effect (dot‐dashed line) and the baseline QTcF0 (dashed line). The circadian rhythm is depicted by the shaded area around the typical profile of QTcF interval.

